# Colitis induced by IL-17A-inhibitors

**DOI:** 10.1007/s12328-023-01893-9

**Published:** 2023-12-07

**Authors:** Lea Grümme, Sophia Dombret, Thomas Knösel, Alla Skapenko, Hendrik Schulze-Koops

**Affiliations:** 1 Division of Rheumatology and Clinical Immunology, Department of Medicine IV, LMU Clinic Munich, Pettenkoferstraße 8a, 80336 Munich, Germany; 2 Institute of Pathology, LMU Clinic Munich, Munich, Germany

**Keywords:** IL-17A-inhibitors, Secukinumab, Ixekizumab, Colitis, Rheumatology

## Abstract

**Background:**

Interleukin (IL)-17A is essential for intestinal mucosal integrity, contributing to the prevention of detrimental immunity such as infectious colitis and inflammatory bowel disease (IBD). Indeed, neutralization of IL-17A has been abandoned as a therapeutic principle in IBD because of increased disease activity. However, it is controversial whether IL-17A inhibitors increase the risk of developing colitis in patients who do not have underlying IBD. Here, we present two cases of different forms of colitis that occurred during treatment with two IL-17A inhibitors, secukinumab and ixekizumab.

**Case presentations:**

We report the case of a 35-year-old female with SAPHO (synovitis–acne–pustulosis–hyperostosis–osteitis) syndrome who was admitted due to severe colitis with bloody diarrhea, fever, abdominal pain and weight loss after receiving secukinumab for 3 months as well as the case of a 41-year-old male with psoriatic arthritis who presented himself to the outpatient clinic with bloody stools, abdominal pain and nausea 5 months after changing his therapy from secukinumab to ixekizumab. In both patients, treatment with IL-17A-inhibitors was stopped and tumor necrosis factor inhibitors were started. Both patients recovered, are clinically stable and show no more signs of active colitis.

**Conclusion:**

The role of IL-17A inhibitors in the pathogenesis of infectious colitis and new-onset IBD is not fully understood and requires further research. Patients receiving IL-17A-inhibitor therapy should be carefully screened and notified of the possible side effects.

## Background

Interleukin (IL)-17A is a proinflammatory cytokine produced by T helper (Th) 17 lymphocytes. Physiologically, Th17 cells are located in the lamina propria of the gastroenterological wall and are essential for maintaining intestinal mucosal integrity, playing a crucial role in mucosal immunity against intestinal pathogens [1–6]. IL-17A facilitates innate immunity by inducting chemokine and inflammatory cytokine production and by secreting antimicrobial peptides such as β-defensins, S100A8 and lipocalin 2 [2–4]. Consequently, IL-17A is particularly important in the immune defence against extracellular pathogenic fungal and bacterial species such as Candida, Cryptococcus, Klebsiella and Staphylococcus [4].

The role of IL-17A in inflammatory bowel disease (IBD) has been extensively studied, but has not been fully understood. Elevated levels of IL-17A und IL-17F have been found in patients with IBD and the increased concentration of IL-17A within the lamina propria of patients with Crohn’s disease suggested a possible therapeutic target. Two-phase II trials testing IL-17A-inhibitor agents in patients with Crohn’s disease were terminated due to increased occurrence of adverse events as well as an increased disease activity in the treatment group compared to the placebo group [7, 8]. Furthermore, there have been several reports of exacerbations in patients with IBD as well as clinical cases of newly diagnosed IBD in patients with no prior history of IBD after receiving treatment with IL-17A-inhibitors [9–13]. Therefore, the potential risk of IL-17A-inhibitors causing colitis is being discussed, especially as the underlying pathophysiological mechanisms are unclear [7, 14]. A recent large retrospective case series reported the alarming number of 850 cases of new-onset IBD and 279 cases of colitis in patients treated with secukinumab and ixekizumab, highlighting the significance of this issue [15].

Although the pathogenesis of psoriatic arthritis (PsA), psoriasis (PsO) and ankylosing spondylitis (AS) is not yet fully understood, IL-17A-mediated immune responses seem to play a central role: The dysregulation of Th17 cells can lead to chronic inflammation and affect various tissues, resulting in joint and skin inflammation and causing diseases such as PsA, PsO and AS [7]. Consequently, the neutralization of increased IL-17A levels has been associated with reduced disease activity in patients with PsA, PsO and AS [16]. Secukinumab and ixekizumab are human and humanized monoclonal antibodies targeting IL-17A, and they are used in the treatment of PsO, PsA and AS [17].

We present two patients who developed episodes of severe gastrointestinal symptoms during therapy with secukinumab and ixekizumab.

## Case presentations

*Case 1* In February 2021, a 35-year-old female, Caucasian patient presented with a 4-week history of bloody diarrhea, abdominal pain and fever reaching 38.1 °C. She had a medical history of SAPHO (synovitis–acne–pustulosis–hyperostosis–osteitis) syndrome and had been receiving off-label secukinumab therapy since October 2020, which has previously been shown to be efficacious in SAPHO syndrome [18]. In addition, she had a history of hypothyroidism and vitamin D deficiency. Her self-medication included L-thyroxine and vitamin D. The patient had no recent travel history and had not taken any other medication before the onset of symptoms. This was the first time she had experienced gastroenterological symptoms, and her family history was negative for IBD.

**Fig. 1:**
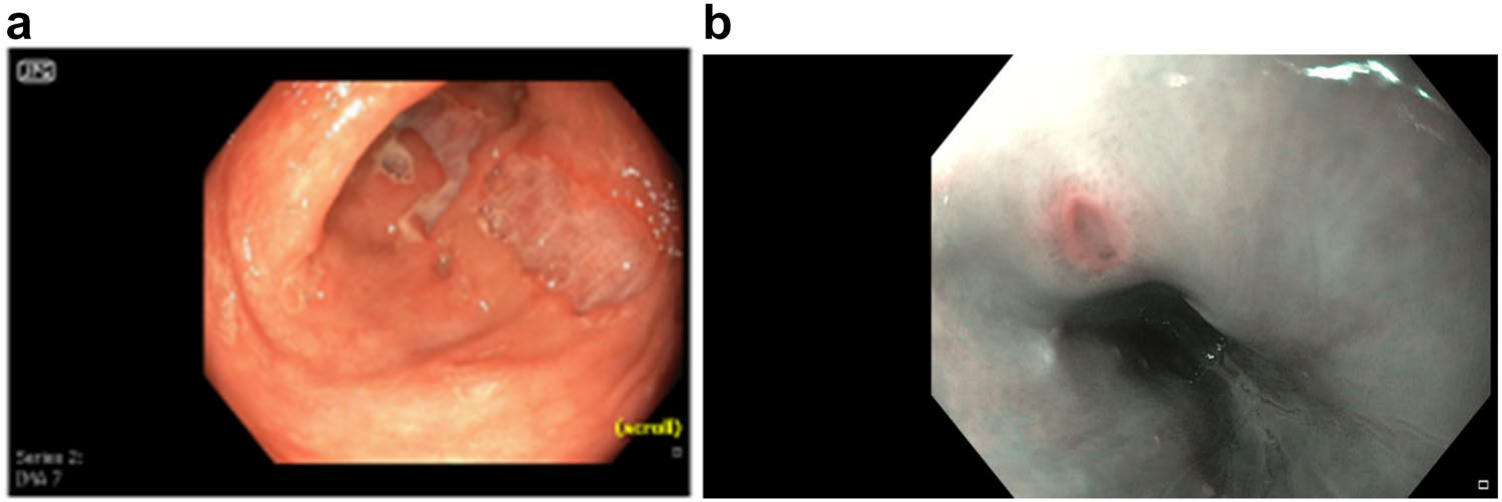
**a** Deep, punched-out mucosal defects in the sigmoid region as well as severely inflamed mucosa with large areal ulcerations. **b** Florid ulcer in the distal esophagus

**Fig. 2:**
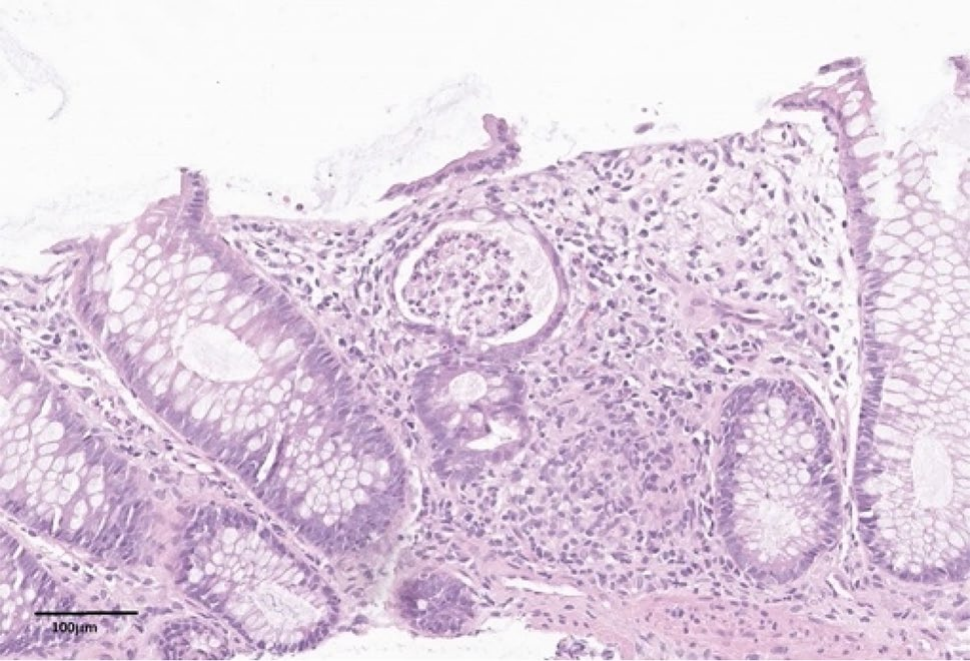
Ulcerated colon mucosa with regenerated epithelium and a crypt abscess (H&E, magnification 20x)

Upon admission, there were no signs of relevant SAPHO-disease activity or a history of chronic non-steroidal anti-inflammatory drug (NSAID) intake. The last dose of secukinumab had been administered in mid-January 2021. Shortly afterwards, she began experiencing bloody diarrhea with a stool frequency of 8–10 times per day, requiring a 1-week inpatient treatment in early February 2021. The patient presented in a reduced general condition but was cardio-respiratory stable. Physical examination revealed regular bowel movements and a soft abdomen. There were no signs of skin lesions or swollen and painful joints. A partial colonoscopy was performed, revealing severe colitis of the left colon with ulcerations, mucosal edema and aphthae (Fig. 1a). Histological results indicated acute inflammation (described as “non-typical for IBD”) (Fig. 2). An endoscopic examination of the upper gastro-intestinal-tract showed an ulceration in the distal esophagus (Fig. 1b). Secukinumab was discontinued and systemic therapy with glucocorticoids and mesalazine was initiated, resulting in a significant improvement in diarrheic episodes and systemic inflammation markers. The patient was discharged with a prescription of oral prednisolone at 60 mg/day, with plan to gradually taper the glucocorticoids until the follow-up outpatient appointment.

After reducing the prednisolone dosage to 50 mg/day, the symptoms worsened, leading to a second hospital admission at the end of February 2021. Upon readmission, the patient reported episodes of bloody diarrhea up to 8 times per day and an unintended weight loss of 6 kg over the last 8 weeks. Laboratory results showed elevated systemic inflammation parameters, with a C-reactive protein (CRP) level of 76 mg/l (reference: ≤ 5 mg/l) and leukocytosis of 17.5 G/l (reference: 4.00–10.40 G/l), as well as an elevated calprotectin level in her stool at 4822 mg/l (reference < 50 mg/l negative, 50–200 mg/l grey area, > 200 mg/l severe inflammation). Microbiological examination of stool cultures ruled out infection with *Clostridium difficile*, *Campylobacter jejuni* or other enteropathogens. There were no findings of an acute or chronic hepatitis B or C, and no indication of an infection with human immunodeficiency virus (HIV), cytomegalovirus (CMV) or herpes simplex virus (HSV).

**Fig. 3:**
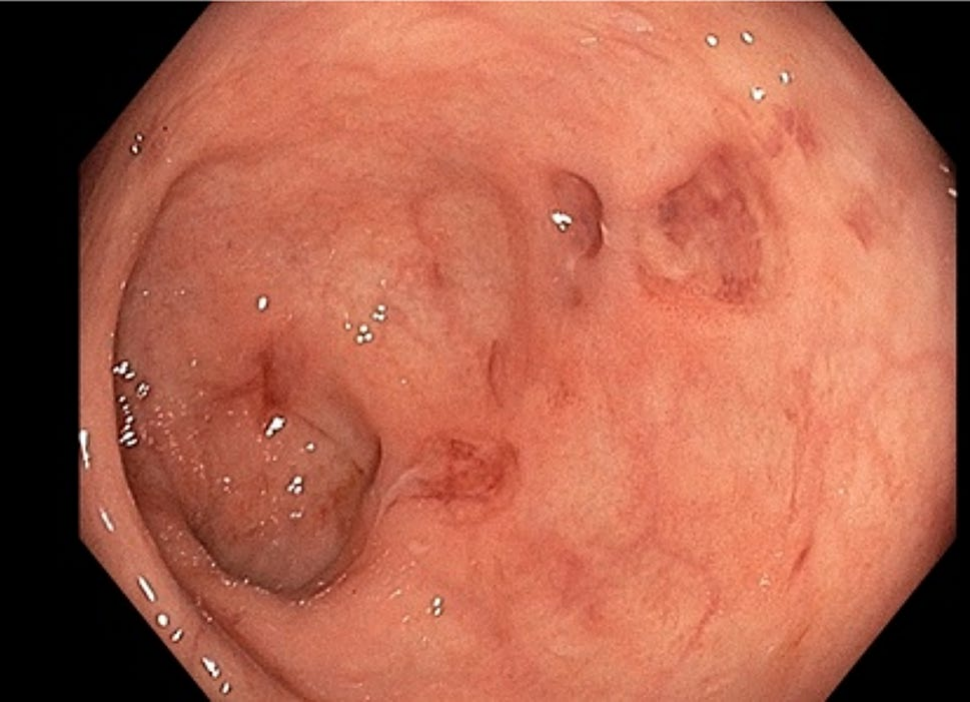
Deep, punched-out mucosal defects in the area of the sigmoid as well as severely inflamed mucosa with sometimes extensive ulcerations

The suspected diagnosis was a possible case of ulcerative colitis induced by secukinumab. The initial microscopic finding did not correlate with the macroscopic image, so another rectosigmoidoscopy was performed. The colonoscopy revealed signs of acute inflammation as well as deep penetrating ulcerations (Fig. 3). The second colonoscopy showed continuous inflammation with deep mucosal defects and fibrin coating in all sections of the colon examined (extending to the area of the left flexure). The defects increased proximally—rather atypical for ulcerative colitis. Histopathology revealed no skip lesion, giant cells, or granuloma, which would have been expected in Crohn’s disease. There was no evidence of specific pathogens. Thus, both macroscopically and histologically, the changes in the colon were not suggestive of any specific disease. In the synopsis of all findings, a toxic drug side effect was most likely to be considered. The esophageal ulceration in the esophagus was considered to be a stress ulceration in the context of the severe clinical picture, although the exact cause of the esophageal ulcer in our patient was not entirely clear, as esophageal ulcers are rarely induced by stress. However, there was no finding of CMV, HIV, HSV or tuberculosis, which commonly cause esophageal ulceration, especially in immunosuppressed patients. The lower-GI mucosal lesions did not fit the typical pattern for Crohn’s disease. Hence, drug-induced esophagitis and ulcerations were considered, although our patients had not taken medications such as doxycycline, bisphosphonates, iron or NSAIDs before the onset of symptoms. To our knowledge, there are no reported cases of IL-17-induced esophageal ulcerations, although we cannot rule out an association. As there were no signs of reflux or esophagitis, these possible causes were considered less likely. Biopsies revealed acute inflammation without signs of malignancy of enteropathogens like lambliasis. Mesalazine treatment was discontinued, as it had been administered for about 3 weeks without significant clinical improvement. Due to the patient’s significantly reduced general condition, rapid anti-inflammatory therapy was necessary. Intravenous prednisolone at 250 mg/day was administered for 4 days, resulting in a favorable clinical response. To prevent secondary bacterial infections, a calculated antibiotic therapy with metronidazole and ciprofloxacin was initiated as a mucosal barrier dysfunction was expected due to the severe inflammation of the colon.

Upon reducing of prednisolone to 60 mg/day, the patient experienced a reoccurrence of symptoms, with an increased frequency of bloody diarrhea. High-dose intravenous prednisolone therapy (250 mg/day) was repeated for 4 days and therapy with a tumor necrosis factor (TNF)-inhibitor, adalimumab, was initiated at an initial dose of 120 mg. With this treatment, the patient gradually showed clinical improvement, with declining inflammatory parameters in the blood as well as fecal calprotectin levels. The prednisolone dosage was reduced to 60 mg/day without further symptom aggravation. Adalimumab was continued at a dosage of 80 mg every 2 weeks. Following discharge from the hospital, the glucocorticoid dosage was subsequently tapered under close clinical observation, along with monitoring of fecal calprotectin levels and inflammation parameters in blood samples. In the follow-up appointments 3 and 6 months after discontinuing secukinumab, the patient remained clinically stable. Notably, clinical symptoms of the SAPHO syndrome did not reoccur. A colonoscopy in May 2021 showed no signs of active inflammation.

**Fig. 4:**
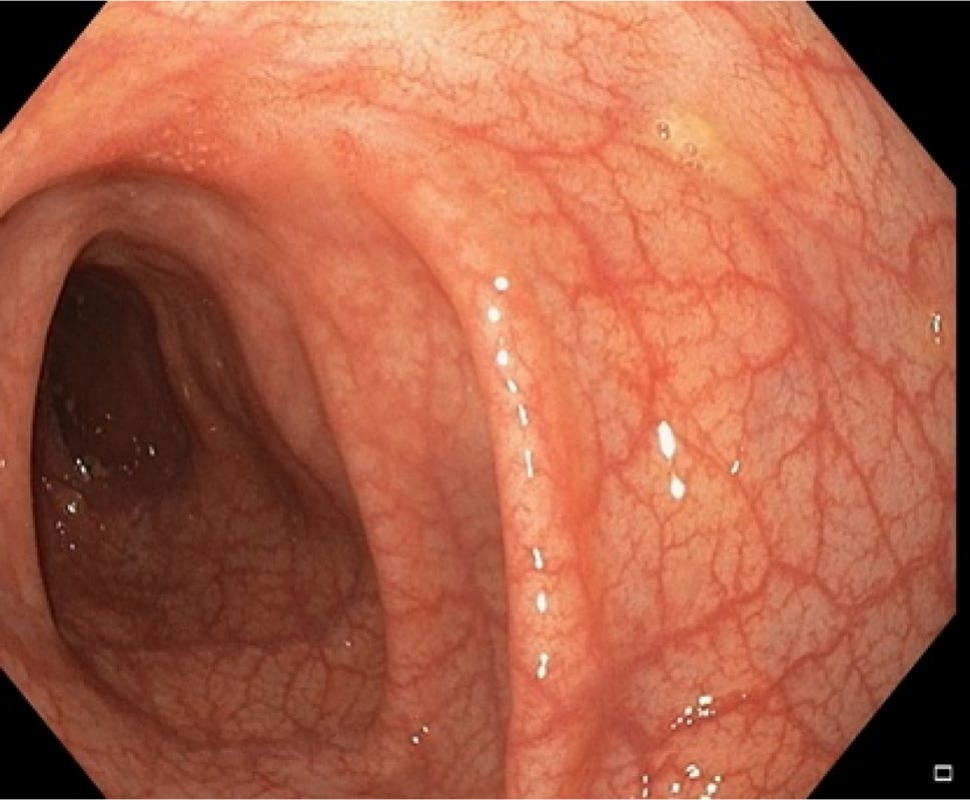
Moderate inflammation activity and ulceration in the left hemi-colon

However, in September 2021, the patient experienced another episode of colitis. A sigmoidoscopy was performed, revealing moderate inflammation activity and ulceration in the left hemi-colon (Fig. 4). No pathogens were detected in the stool diagnostic workup. Fecal calprotectin was measured at 1273 mg/l and the CRP levels in the blood were 17 mg/l. Screening for CMV and Epstein Barr virus was negative. Assuming a secondary loss of efficacy, the anti-inflammatory therapy was changed from adalimumab to golimumab (200 mg as a loading dose, 100 mg every month as a maintenance therapy) and a temporary therapy with prednisolone was initiated. At the time of the follow-up appointment, the patient reported that she was feeling much better. There was no longer diarrhea or bloody stool. The abdominal pain had improved significantly. Adalimumab anti-drug antibodies were positive (13.6 arbitrary units (AU)/ml, reference < 10 AU/ml). Since July 2022, the patient has been in complete clinical remission with respect to IBD and SAPHO, receiving 100 mg of golimumab every month and no other immunosuppressive medication.

**Fig. 5:**
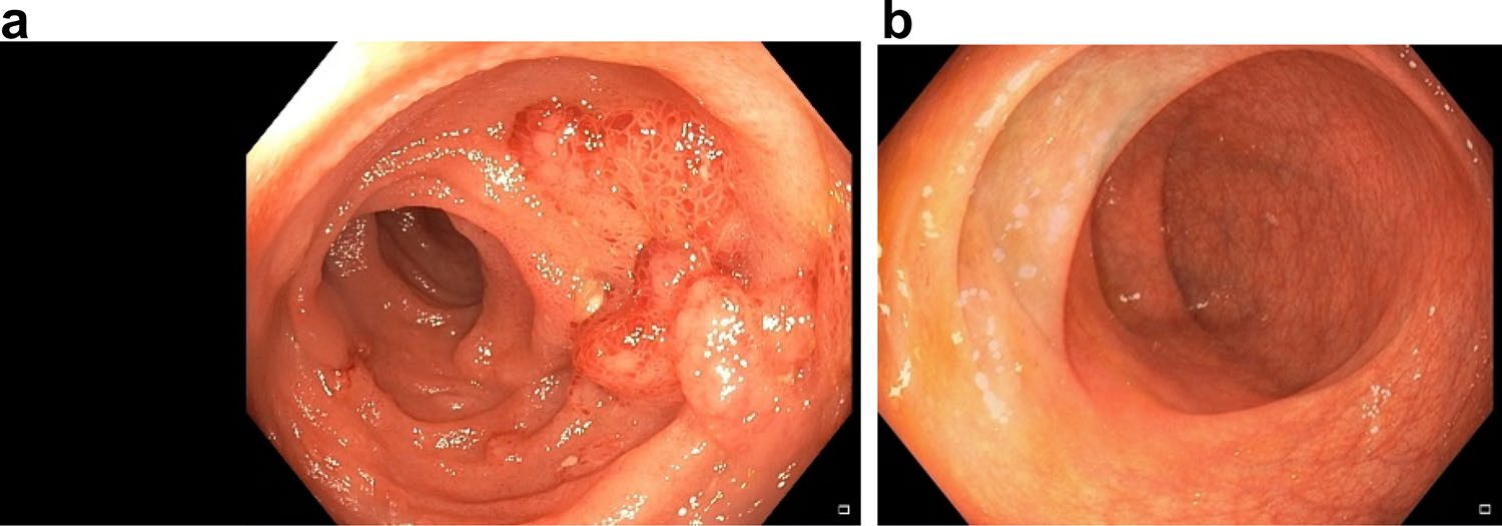
**a** Unclassifiable colitis and ileitis with segmentally distributed aphthae and redness. Partially the mucosa appears edematous. **b** Colonic mucosa without significant abnormalities

**Fig. 6:**
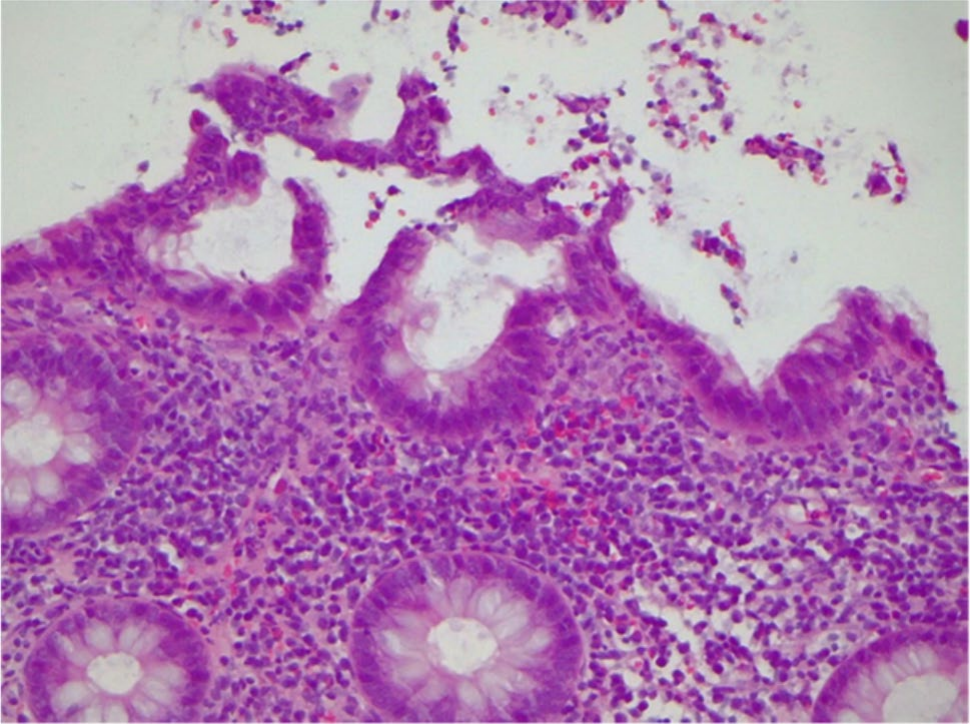
Colonic mucosa with apical pseudopapillary changes and intraepithelial neutrophils (magnification 200×)

*Case 2* In February 2021, a 41-year-old Caucasian male, who was in rheumatological care for PsA since 2016, presented for a regular check-up in our outpatient clinic. He had no further pre-existing conditions. For his PsA, he had already been treated with methotrexate, golimumab (for 1 year, discontinued due to secondary loss of efficacy) and secukinumab (for about 3 years, discontinued because of secondary loss of efficacy). Five months prior, a therapy with ixekizumab 80 mg per month had been started. Initially, the patient tolerated the therapy well. For the past 6–7 weeks, however, he has had bloody stools, abdominal pain, nausea after food and fluid intake, and occasional tarry stools. He did not have any mucous deposits nor did he have a family history of IBD. The PsO lesions of the skin and the axial complaints had also increased during the weeks prior to his appointment. There was no history of recent travel. The patient presented in stable general condition and normal nutritional status. Physical examination revealed regular bowel movements and a soft abdomen, however with diffuse pressure sensitivity. The patient reported pain on percussion of the lumbar spine. In the joint examination 11 joints were painful, none of them swollen. Psoriatic lesions existed on the abdomen and navel, the scalp, the hands and arms on both sides. Laboratory chemistry showed that the patient had discreetly elevated parameters of systemic inflammation (CRP 7 mg/l) and slightly elevated liver enzymes (aspartate aminotransferase (GPT/ALT): 53 U/l (reference: ≤ 49 U/l), gamma-glutamyltransferase (gamma-GT) 91 U/l (reference: ≤ 59 U/l)). Stool diagnostics were negative for clostridium difficile toxin and campylobacter as well as for other bacterial and viral enteropathogens and microscopically for worm eggs, lamblia, amoeba, and cryptosporidia. Gastrointestinal endoscopy revealed colitis and ileitis that could not be further classified showing segmentally distributed aphthae and redness (Fig. 5). In part, the mucosa appeared edematous. Multiple ulcerations were detectable in the coecum. Histology showed inflammatory activity with microcrypts and a regenerative structured mucosa, suggestive for infectious colitis (Fig. 6). IBD could not be confirmed, and there was no evidence of dysplasia, malignancy or CMV infection. Upper GI-endoscopy was performed and revealed reflux polyps. Histologically, a chemical-reactive antrum gastritis could be detected without signs of dysplasia, malignancy, Barrett’s mucosa or helicobacter pylori. As no infectious enteropathogens were detected and the macroscopic findings were not suitable for infectious colitis, an infection cause seemed not likely and the patient received no specific treatment in this regard. Due to the macroscopic pattern of the colitis as well as in overall view of the constellation a drug-induced colitis could not be ruled out. In consultation with the gastroenterologist, we discontinued the therapy with ixekizumab and started adalimumab at a dose of 40 mg subcutaneously every 2 weeks. Approximately 8 weeks after initiation of therapy, the patient presented to the gastroenterology department with improved, but persistent gastrointestinal symptoms. He reported recurrent bloody diarrhea with approximately 3 stools daily. Serologically, no evidence of acute, chronic, or latent hepatitis B or C could be found. Again, stool samples were taken but they still could not detect any pathogen. An increased calprotectin level was detected (314 mg/l). Despite the continued lack of pathogen detection, but in view of the histological findings in the colonoscopy suggestive for infectious colitis and the ongoing gastroenterological symptoms, a probationary antibiotic therapy with metronidazole 400 mg t.i.d. and ciprofloxacin 500 mg b.i.d. for a total of 5 days was started. Three weeks later, the patient presented in a much improved condition. He had normal bowel movements up to 3 times per day. No bloody stools or abdominal pain were reported. In parallel, the PsO lesions of the skin as well as the joint complaints were regressive. A re-colonoscopy was not performed due to the significant improvement in symptoms. Currently, the patient is treated with adalimumab as per label and he is in constant symptom-free remission.

## Discussion

IL-17A plays a pivotal role in maintaining intestinal mucosal homeostasis. On the one hand, it contributes to mucosal immunity against intestinal pathogens. Blocking IL-17A could, therefore, increase the risk of infectious colitis. On the other hand, it prevents autoimmune and autoinflammatory immune responses and thus mitigating the development of IBD. Nevertheless, IL-17A blockade is highly efficient in a number of rheumatologic and dermatologic diseases, such as PsO, PsA, and AS. Our case observations underscore the risk of colitis, including both autoimmune and potentially infectious forms, in patients undergoing IL-17A-inhibitor therapy. The severity of intestinal inflammation can be substantial and should not be underestimated.

In the first patient, who had a history of SAPHO syndrome, severe colitis developed after 3 months of exposure to the IL-17A inhibitor. This patient had no prior history of IBD and lacked known risk factors (e.g., negative family history and no smoking history). Gastrointestinal symptoms were absent before secukinumab treatment commenced. Extensive diagnostic evaluation did not reveal any other potential causes for these clinical symptoms. An association between IBD and other autoimmune disorders such as thyroiditis [19], celiac disease [14], multiple sclerosis [19], and psoriasis [20] has been reported [21]. The prevalence of IBD in patients with SAPHO syndrome has been estimated to range from 5% [21] to up to 8 to 13% in two previous studies [22, 23]. Therefore, it appears likely that the patient’s baseline risk for IBD was elevated compared to the general population, regardless of exposure to secukinumab.

The question of whether patients treated with secukinumab have a higher risk of developing IBD remains controversially debated [8]. A comparative study indicated that patients with PsA/AS, initiating IL-17A-inhibitor therapy do not have a higher risk of developing IBD when compared to patients initiating etanercept [24]. However, similar cases of new-onset colitis during IL-17A-inhibitor therapy [9, 10, 12, 13, 15] and data suggesting paradoxical colitis activity in Crohn’s disease patients [8] raise concerns. Given the typical overall presentation of the case as well as the new onset of symptoms after recent exposure to secukinumab, a drug-induced etiology of IBD seemed plausible.

In the second patient, the pathogenesis of colitis appears less clear. Possible differential diagnoses include infectious and/or autoimmune colitis, and both underlying pathomechanisms may be favored under IL-17A-inhibitor therapy.

IL-17A's role in maintaining intestinal mucosal homeostasis is multifaceted. In animal models, blocking IL-17A has led to increased intestinal inflammation and compromised epithelial barriers [7, 10]. Multiple studies have shown that IL-17A is particularly crucial for the immune response against extracellular pathogenic fungal and bacterial species [2–5]. In a mouse model, IL-17 receptor E-deficient mice exhibited not only increased colon pathology and bacterial load during infection with *Citrobacter rodentium*, but also significantly decreased expression of genes encoding antibacterial peptides and other inflammatory molecules [6]. This resulted in a significantly increased mortality rate among IL-17 receptor E-deficient mice [6]. IL-17F and IL-17A also play essential roles in the immune defence via activation of cytokines of the intestinal epithelium [5]. IL-17A is predominantly produced in T cells, while IL-17F is produced in T cells as well as in cells of the innate immune system, and epithelial cells. Both IL-17F and IL-17A are essential for immune defence against *Staphylococcus aureus* and *Citrobacter rodentium* [5]. Therefore, there might be an increased risk for infectious colitis during IL-17A-inhibitor therapy. In our patient, macroscopic findings suggested IBD, while the microscopic findings from endoscopic examinations suggested primarily infectious colitis. The patient’s symptoms improved after discontinuation of ixekizumab and therapy with adalimumab. However, as some symptoms persisted, the patient received antibiotic therapy (even though no enteropathogens were detected in an extensive diagnostic search). Eventually, the patient’s symptoms disappeared. Whether the patient recovered due to the antibiotic treatment remains unclear. Several studies have shown that antibiotics cannot only treat bacterial infections but also have a significant immune-modulating impact [25–27]. An improvement in the patient’s symptoms after antibiotic therapy does not rule out an autoimmune cause of the symptoms, possibly triggered by the switch in therapy from secukinumab to ixekizumab. Another potential reason might be that an infectious cause, due to an impaired barrier function, contributed to the symptoms. In IBD, impaired epithelial barrier function and increased pathogen accessibility to the intestinal mucosa are common phenomena [28].

The switch from secukinumab to ixekizumab in our patient’s case is notable. In the presented case, the medication was not switched to ixekizumab due to side effects but due to a secondary loss of efficacy. The patient had been treated with secukinumab for several years without developing gastrointestinal symptoms. However, about 6–7 weeks after starting ixekizumab therapy, severe signs of colitis emerged. To the best of our knowledge, there are no reported cases of patients developing colitis after switching therapy from one IL-17A inhibitor to another, such as from secukinumab to ixekizumab or vice versa. In contrast, several studies have demonstrated the safety of switching from secukinumab to ixekizumab [29, 30]. This seems especially useful in difficult to treat patients, who previously failed to respond to secukinumab therapy [29, 30]. The reason for the development of colitis after switching from secukinumab to ixekizumab remains unclear [20]. Even though secukinumab and ixekizumab are both IL-17A-inhibitors, several studies have tried to demonstrate differences between the two drugs [31, 32]. Secukinumab has a longer half-life and is administered at a dosage 3–4 times higher than that of ixekizumab, with peak efficacy occurring approximately 16 weeks after initiation [31]. This results in significantly greater systemic exposure [31]. Ixekizumab has significant higher affinity to IL-17A and IL-17A/F [29, 31]. The increased affinity could account for the lower dosage needed compared to secukinumab [31]. Ixekizumab achieves peak efficacy approximately 12 weeks after initiation [31]. It is clear, that despite both being IL-17A-inhibitors, secukinumab and ixekizumab have distinct pharmacokinetic characteristics [31]. These differences in affinity, specificity, and systemic exposure may significantly impact side effects, and efficacy [29].

In our case, the different affinity to IL-17A and IL-17A/F is particularly interesting, given that IL-17A/F heterodimers and IL-17A homodimers signal through the same IL-17 receptor A/receptor C complex (IL-17RA/RC) [29, 33]. Ixekizumab has higher affinity to both IL-17A and IL-17A/F. The IL-17A/F heterodimer is a cytokine, which mimics IL-17A as well as IL-17F [29, 33]. IL-17A and IL-17F are proinflammatory cytokines that are elevated in patients with IBD. This pharmacological characteristic may contribute to the efficacy of ixekizumab in patients who did respond to secukinumab [29]. Moreover, these differences in affinity could also explain the severe gastrointestinal symptoms experienced by our patient after being switched from secukinumab to ixekizumab. However, a recent databank analysis found that the number of IBD cases that occurred after secukinumab treatment was 10 times greater than that observed after ixekizumab therapy [34]. The authors mention that the number of IBD cases among patients treated with secukinumab and ixekizumab could, in part, be traced back to the different launch dates (January 2015, April 2016) [34].

Possibly, anti-drug antibodies neutralizing the effect of secukinumab might explain the absence of gastrointestinal symptoms during secukinumab treatment and their emergence with ixekizumab. The patient’s treatment was changed from secukinumab to ixekizumab due to a secondary loss of efficacy. Anti-secukinumab-antibodies could have neutralized the effect of secukinumab, preventing the triggering of IBD.

In clinical practice, patients experiencing IL-17 inhibitor-induced IBD typically present with symptoms such as diarrhea, bloody stools, abdominal pain, and fever. These symptoms are often accompanied by elevated white blood cell count, erythrocyte sedimentation rate, C-reactive protein levels, and fecal calprotectin concentration [34]. The majority of new-onset IBD cases have been detected within three months of commencing anti-IL-17 therapy [34]. Individuals with a history of gastrointestinal symptoms or prior colitis episodes, regardless of the underlying autoimmune condition, may be at higher risk [11]. Screening measures such as assessing prior gastrointestinal symptoms and family history of IBD are beneficial, and non-invasive biomarkers like fecal calprotectin may enhance IBD risk assessment.

Currently, there is no clinical guidance for the management of patients with disease flares or new-onset IBD after IL-17 inhibitor therapy [34]. Nevertheless, discontinuation of IL-17 inhibitors has reportedly lead to a substantial improvement of symptoms among patients with IL-17A inhibitor-associated colitis [34]. In the majority of cases, appropriate management has led to symptom resolution within 4 weeks [34].

While IL-17 inhibitors are generally considered safe and exhibit high efficacy in managing PsO, PsA, and AS, it is important to note that rare adverse effects have been documented. These may include new-onset or exacerbation of IBD, although a definitive causal link has yet to be established. Larger prospective studies could help to better understand of the connection between IBD and IL-17 inhibition and the frequency of occurrence of this rare adverse event [11].

## Conclusion

In conclusion, the role of the IL-17A-inhibitors in the development of colitis remains not fully understood and requires further investigation. Given the significant role of IL-17A in the homeostasis of the intestinal mucosa and the potential risk of infectious colitis and new-onset IBD under IL-17A-inhibitor therapy, it is crucial for healthcare providers to carefully select patients for this treatment and inform them about the potential side effects. This may be especially important when considering the commonly performed switch from secukinumab to ixekizumab in difficult-to-treat patients. Inquiring about prior occurrences of gastrointestinal symptoms and investigating family history for IBD prior to treatment with IL-17-inhibitors might be a useful screening measure. Faecal calprotectin is a non-invasive, widely available biomarker, commonly used to monitor IBD disease activity. In line with previous authors [15], we believe it might be a suitable tool to further improve screening for IBD risk patients.

## Abbreviations

ALT: Aspartate aminotransferase
AU: Arbitrary units
AS: Ankylosing spondyloarthritis
b.i.d.: Two times a day
CMV: Cytomegalovirus
CRP: C-reactive protein
dl: Deciliter
DNA: Deoxyribonucleic acid
EBV: Epstein–Barr virus
ELISA: Enzyme-linked immunoassay
G: Giga
Gamma-GT: Gamma-glutamyltransferase
IBD: Inflammatory bowel disease
HIV: Human immunodeficiency virus
HSV: Herpes simplex virus
IL: Interleukin
kg: Kilogram
l: Liter
mg: Milligram
ml: Milliliter
NSAID: Non-steroidal anti-inflammatory drugs
PsA: Psoriasis arthritis
PsO: Psoriasis
SAPHO: Synovitis–acne–pustulosis–hyperostosis–osteitis
Th: T helper
t.i.d.: Three times a day
TNF: Tumor necrosis factor
